# On the Improvement of Thermal Protection for Temperature-Responsive Protective Clothing Incorporated with Shape Memory Alloy

**DOI:** 10.3390/ma11101932

**Published:** 2018-10-10

**Authors:** Jiazhen He, Yehu Lu, Lijun Wang, Nini Ma

**Affiliations:** 1 National Engineering Laboratory for Modern Silk, Soochow University, Suzhou 215000, China; jzhe@suda.edu.cn; 2 College of Textile and Clothing Engineering, Soochow University, Suzhou 215000, China; wanglijun1027@outlook.com (L.W.); 20164215039@stu.suda.edu.cn (N.M.)

**Keywords:** shape memory alloy, thermal protective clothing, thermal insulation, air gap

## Abstract

This study explored the application of shape memory alloy (SMA) springs in a multilayer protective fabric assembly for intelligent insulation that responded to thermal environment changes. Once the SMA spring was actuated, clothing layers were separated, creating an adjustable air gap between the adjacent fabric layers. The impacts of six different SMA arrangement modes and two different spring sizes on thermal protection against either a radiant heat exposure (12 kW/m^2^) or a hot surface exposure (400 °C) were investigated. The findings showed that the incorporation of SMA springs into the fabric assembly improved the thermal protection, but the extent to which the springs provided thermal protection was dependent on the arrangement mode and spring size. The effectiveness of reinforcing the protective performance using SMA springs depended on the ability of clothing layers to expand an air layer. The regression models were established to quantitatively assess the relationship between the air gap formed by SMA spring and the thermal protective performance of clothing. This study demonstrated the potential of SMA spring as a suitable material for the development of intelligent garments to provide additional thermal protection and thus reduce the number of clothing layers for transitional thermal protective clothing.

## 1. Introduction

Thermal hazards are common dangers faced by firefighters, industrial workers in the metallurgical and energy industry, military personnel, and race car drivers. Intense heat transfer from the thermal environment to human skin may result in severe skin burn injuries. Prevention of skin burn injuries associated with exposure to thermal hazards has long been identified as the primary function of thermal protective clothing (TPC) [1]. It is reported that there were an estimated 29,130 injuries to firefighters during fire ground operations in the U.S. in 2015 [2]. Therefore, TPC with excellent thermal protective performance is essential to protect the body from external heat exposure hazards.

To improve the thermal protective performance of clothing, new types of textile fibers need to be developed, as they are the basic raw materials needed to manufacture clothing. Adding layers or thickness to fabrics is also an effective way to improve the thermal insulation of protective clothing [3,4], thereby decreasing the heat transfer through thermal environments. However, clothing weight and bulkiness add additional physiological burden that can lead to heat stress problems [5,6] and induce discomfort in stressful conditions typically associated with occupations such as firefighting [7]. Multilayered and thick fabrics can also store a lot of thermal energy during exposure, which may result in great heat discharge and the occurrence of after-exposure skin burns [8,9]. In recent years, incorporating aerogel [10,11] and phase change materials (PCMs) [12,13] into TPC have interested researchers as these materials can improve the thermal protection provided by traditional protective clothing. Despite their notable advantages, these systems have several limitations, including the large stiffness and high evaporative resistance caused by the aerogel, and the burning behavior, durability problems, increasing evaporative resistance and clothing weight caused by the PCMs [14].

Ideal TPC should provide adequate thermal insulation only when thermally challenged by heat and flame, and offer less thermal insulation concordant with comfort requirements under normal conditions. This goal can be achieved by introducing an expanding air gap between the layers of TPC when the temperature changes. The use of shape memory alloys (SMAs) is a novel method to meet this requirement [15,16]. SMAs have been used as actuators in many mechanical processes for some time [17]. The alloys are trained to return to a given shape when an actuation temperature is reached. The early work related to the incorporation of SMA into TPC was conducted by Congalton [18]. A SMA flat conical spring with an actuation temperature of 50 °C was incorporated into a three-layer fabric assembly, and when the thermal challenge occurred, the spring was activated to form a substantial, insulating air gap between the fabric layers. It was found that the application of SMA has shown good potential to improve the thermal protective performance of clothing. Yates [19] also studied the performance of TPC with SMA stitched in the clothing pockets. In this study, one wire of shape memory material was fashioned into two conjoined loops held in the center by a clip, forming a butterfly shape. It was demonstrated that the SMA showed a significant improvement in performance than the traditional clothing without the SMA. Park et al. [20] analyzed the thermal protective performance of protective clothing when four different attachment methods and two different sewing methods of SMA were applied. The results showed that the attachment methods had limited impact on the thermal insulation and a wave type stitch was better than a square one when SMA springs were attached onto the intelligent turnout gear for firefighters. Recently, Ma et al. [21] investigated the thermal protection of fireproof fabrics with SMA springs under hot surface contact and demonstrated the effectiveness of such a dynamic adaptive structure in improving thermal protection of fabric systems. 

Taken together, these results show the enormous potential of SMA as a suitable material for additional protection in TPC. However, there has been little agreement on the effects of SMA arrangement mode and size. Does the thermal protective performance increase in absolute terms as the number of SMA rises? Or does SMA size have a significant effect on the improvement of thermal protection? The answers to these questions will help in the design of suitable SMA to improve the thermal protective performance of clothing. Since thermal hazard attacks and skin burn injuries are frequently reported by firefighters, an investigation was made on firefighter’s protective clothing in this study. SMA springs were incorporated into the selected fabric assemblies as representatives of firefighter turnout suit materials. The effects of SMA arrangement mode and size on the thermal insulating properties of fabrics were explored and the behaviors under two thermal conditions (radiant heat exposure and hot surface contact) were studied. The goal of this study was to reduce severe burn injuries experienced by firefighters in this and similar occupations by gaining a better understanding of the mechanisms underlying heat transfer when SMA springs are incorporated. The research findings might also provide new insights into the development of temperature-adaptable protective clothing.

## 2. Materials and Methods 

### 2.1. SMA Springs

**Figure 1:**
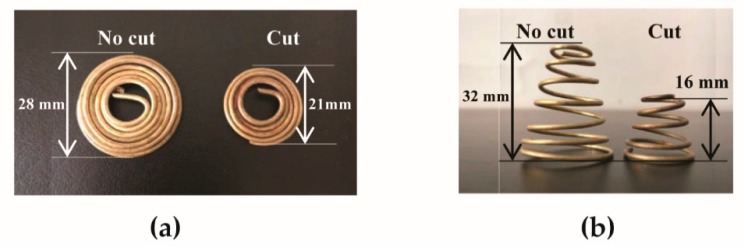
Two sizes of SMA spring at: (**a**) low temperature; (**b**) high temperature.

In this study, SMA springs, made of a copper-based alloy wire (1.5 mm diameter), were developed. This copper-based alloy is one of the main types of SMAs [18] and is relatively cheap to use, making the temperature-responsive protective clothing economical to produce. Each spring was a flat coil at low temperatures (Figure 1a), and quickly changed to a cone shape when the temperature was above 45 °C (Figure 1b). The effectiveness of reinforcing the firefighter protective clothing using the SMA springs should depend on the ability of the protective clothing to expand and form an air layer. It has been reported that 90% air gap size observed for single layer protective clothing is less than 40 mm [22,23]. Moreover, Congalton et al. [18] suggested that air gap size introduced by the SMA should be lower than 35 mm. Based on the above reported facts, the maximum height of the spring after full deformation should be controlled under 35 mm. In this study, two sizes of SMA spring (code-named “No-cut” and “Cut”) were used, and their full deformed heights approximated 32 mm and 16 mm, respectively. The No-cut coil had an outer diameter of 28 mm and an inner diameter of 14 mm, and the Cut one had an outer diameter of 21 mm and an inner diameter of 14 mm. The weight of each No-cut spring was 5 g and the weight of the Cut one was 2.2 g.

### 2.2. Design of Fabric Assemblies with SMA Springs

**Figure 2:**
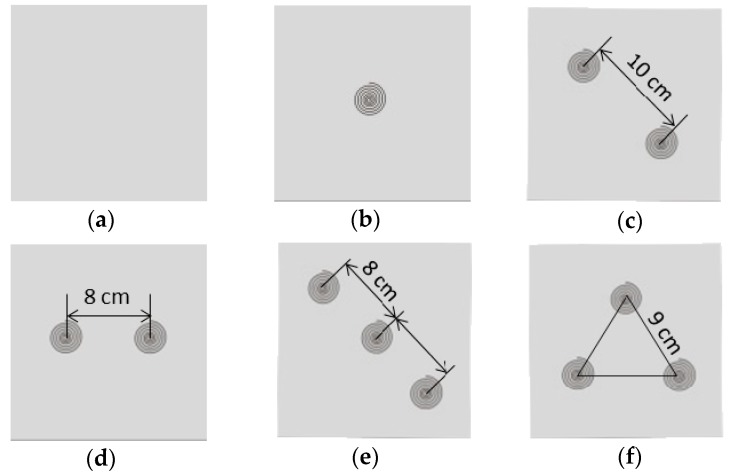
Six different arrangement modes of SMA spring: (**a**) CON: control group without any spring; (**b**) One: one spring at the center; (**c**) Two Diag: two springs positioned diagonally; (**d**) Two Para: two springs positioned in parallel; (**e**) Three Diag: three springs positioned diagonally; (**f**) Three Tria: three springs arranged at the vertices of an equilateral triangle.

Firefighter turnout gear typically consists of a flame-resistant outer shell, a moisture barrier, and a thermal liner. The detailed specifications of the selected fabrics within these layers are displayed in Table 1. The testing fabric assembly in this study was 15 cm × 15 cm and placed with an overlying order from above of a thermal liner, a moisture barrier, and an outer shell. The SMA springs were sewn between the moisture barrier and the thermal liner by using thermal resistance threads to form an air gap between these two fabric layers when the deformation of coil was activated. Six different arrangement modes of the SMA spring were tested (refers to Figure 2): (a) CON—the control group with no spring; (b) One—one spring was located at the center of the fabric specimen; (c) Two Diag—two springs were positioned diagonally, having a 10 cm distance (approximated half the fabric’s diagonal) between them; (d) Two Para—two springs were paralleled in the central line of the fabric and the space between them approximated half the length of the fabric (8 cm); (e) Three Diag—three springs were positioned diagonally. One was in the center and the distance to the other two springs was 8 cm; (f) Three Tria—three springs arranged at the vertices of an equilateral triangle. The distance between each spring was 9 cm. Since heat transfer through the central point of a fabric would be measured in the following thermal protective performance tests, the One and the Three Diag with a spring exactly located in the corresponding measuring point were further selected to be treated with different SMA spring sizes. In addition, the Two Diag that had a similar spring layout with the Three Diag was also selected to examine the effect of the spring size. That is, the One, Two Diag and Three Diag not only differed by spring arrangement modes, but also had different spring sizes (No-cut and Cut). 

### 2.3. Test Conditions and Protocols

Protective performance of the fabric assembly with SMA springs was evaluated by bench-scale tests. Fabric assemblies with SMA springs incorporated within them were preconditioned for at least 24 h in a standard climatic chamber of 20 ± 2 °C and 65 ± 4% relative humidity prior to the test. To determine the thermal protective performance of fabric assemblies under exposures to thermal hazards, a set of thermocouples was installed at fabric locations to measure the temperature distribution through fabric layers. Type-T thermocouples (Omega Engineering, Norwalk, CT, USA; accuracy: ±0.5 °C) with a wire diameter of 0.274 mm were used. Thermocouples locations and their attachment to protective fabrics were determined according to the research of Keiser et al. [24]. Two thermocouples were placed at the center of the external surface of the moisture barrier and the thermal liner, respectively. Another thermocouple was placed at the center of internal surface of the thermal liner. After fabric assemblies were prepared with thermocouples, three fabric layers were sewn together at one pair of the diagonal corners of the fabric assembly to simulate the restriction force caused by the stitches of clothing, or the fabric deformation due to body movement [21]. Then either a radiant heat exposure or a hot surface contact was simulated to perform the thermal protective performance tests. 

• Radiant heat exposures (RHE)

**Figure 3:**
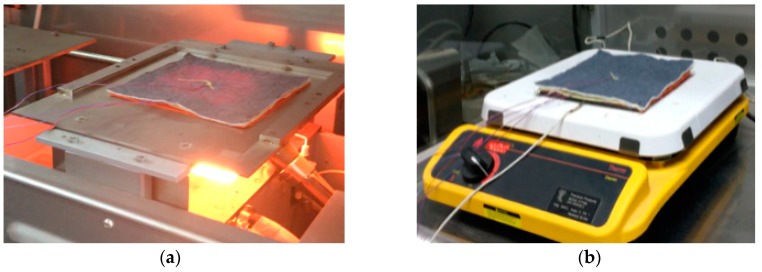
Two different thermal exposure tests: (**a**) RHE: radiant heat exposure test; (**b**) HSC: hot surface contact test.

To perform the laboratory simulation of RHE, the thermal protective performance (TPP) tester (Mode 701-D-163-1, Precision Products LLC, Richmond, VA, USA) was employed (Figure 3a). In this method, radiant heat was generated by a bank of nine translucent quartz infrared lamps placed horizontally beneath a specimen. A produced heat flux of 12 ± 0.3 kW/m^2^ was designed to simulate the low level RHE that is frequently encountered by firefighters [4]. The RHE duration was determined after preliminary experiment, which was set as 70 s to ensure the temperature on the internal surface of the thermal liner could reach 44 °C (see below). 

• Hot surface contacts (HSC)

The TPP of fabrics in HSC exposure was measured according to a modified ASTM F 1060 (West Conshohocken, PA, USA) shown in Sumit’s research [25] (Figure 3b). The specimen of the fabric assembly was horizontally placed in contact with a hot surface plate of electrolytic copper (Precision Products LLC, Richmond, VA, USA). The temperature of the hot surface was controlled at 400 °C. The exposure duration of the fabric was set at 20 s. 

During either a RHE test or a HSC test, the external surface of the outer shell was subjected to thermal exposures (see Figure 3). The data from the thermocouples were collected throughout the exposure duration by using a data acquisition system (National Instruments, NI 9213, Austin, TX, USA). The sampling rate of the data acquisition system was two samples per second for temperature measurements. Three samples of each fabric assembly were tested. The sequence of test specimens with different arrangement modes and SMA spring sizes was randomized.

### 2.4. TPP Analysis Method

Except for the evaluation of temperature histories obtained by thermocouples, the TPP of the fabric assembly under exposures to RHE and HSC was also determined by using the time to reach a sensor temperature rise of 12 °C or 24 °C in accordance with ISO 6942:2002 [26]. The temperature on the internal surface of the thermal liner was used to calculate the time t_44_ and t_56_, i.e., the time to reach the temperature of 44 °C and 56 °C, respectively. In addition, the final temperature throughout the test (T_fa_) was also examined. Therefore, the thermal insulating properties of the fabric assembly with different SMA springs were especially compared by these three indices. 

### 2.5. Statistical Analysis

Descriptive statistics (means and standard deviations) were calculated for all dependent variables: t_44_, t_56_ and T_fa_. All the statistical analyses were processed using SPSS 21.0 software (SPSS Inc., Chicago, IL, USA). A one-way analysis of variance (ANOVA) was used to explore differences of the dependent variables due to the spring arrangement or the spring size. Post hoc analyses were performed using a least significant difference (LSD) test to assess the parameters that displayed significant differences in the ANOVA analysis. 

In the discussion section, fitting analyses were used to derive the correlations between the size of the open air gap and the TPP of the fabric assembly. We were trying to use the linear function, polynomial function, power function and exponential function to conduct the fitting analyses, because these regression functions have been successfully used to establish the relationships between TPP and other independent variables, e.g., fabric weight, fabric thickness, fabric air permeability, and absorbed energy of the skin [23,27]. The goodness of the fitting was examined by using the coefficient of determination (R^2^) and the best fitting equation was determined as the R^2^ was higher than 0.7.

## 3. Results

### 3.1. Performance under RHE

In this section, temperature profiles obtained for the No-cut SMA springs were presented first as examples of how heat transfers through the fabric assembly under the RHE condition. The effects of the SMA arrangement mode as well as the SMA size on TPP under the RHE condition were analyzed thereafter.

#### 3.1.1. Temperature Profiles under RHE

**Figure 4:**
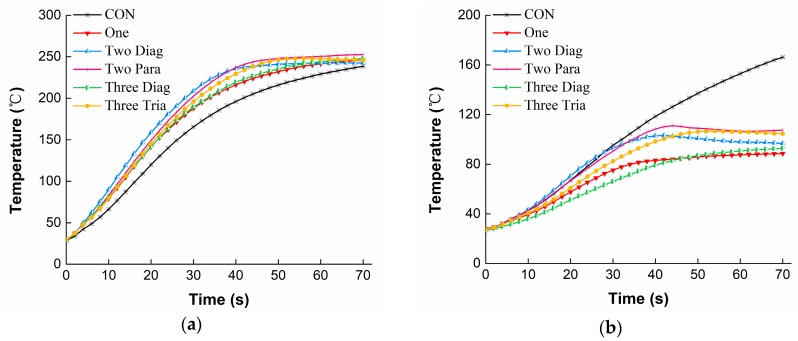

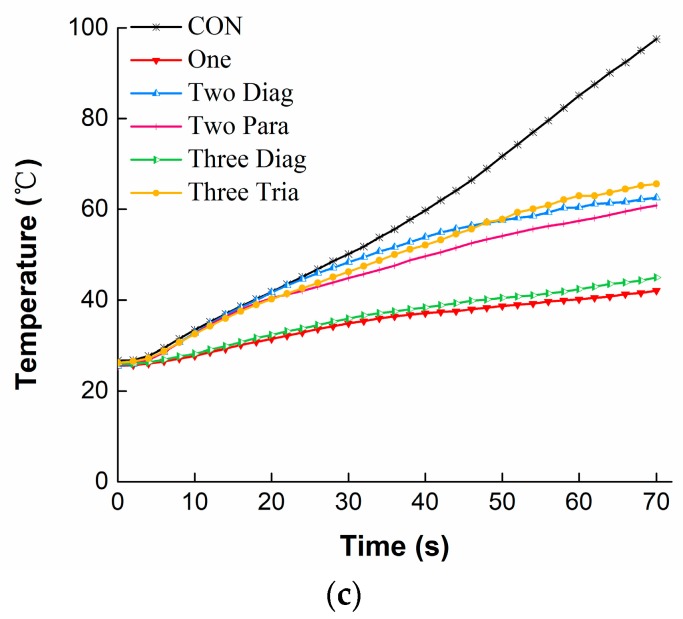
Temperatures for different arrangements of No-cut SMA spring under RHE: (**a**) The external surface of the moisture barrier; (**b**) The external surface of the thermal liner; (**c**) The internal surface of the thermal liner.

Figure 4 shows the temperature distribution through the fabric assembly with different arrangements of No-cut SMA springs under the RHE condition. For the temperature on the external surface of the moisture barrier (Figure 4a), all the curves representing the different SMA arrangements exhibited similar change trends. The temperature increased quickly during the first 40 s of exposure, and then increased very slowly as the exposure continued, showing a final temperature in the range of 235–260 °C. Please note that the temperatures observed for the fabric assemblies with SMA springs incorporated within them were always higher than those without SMA springs (i.e., CON). For the temperature on the external surface of the thermal liner (Figure 4b), a continuous rise was observed for the CON. However, slower increases or even quasi-steady states were observed for the fabric assemblies that had SMA springs incorporated within them after 45 s of exposure. In comparison to the temperatures on the external surfaces of the fabric layers, the temperature on the internal surface of the thermal liner increased slowly and the change trends could be classified into three groups: high, medium, and low levels (Figure 4c). The highest temperature level was observed for the CON, showing 98 °C at the end of the exposure, whereas the lowest temperature level was found for the One and the Three Diag (ending temperature of approximately 43 °C). The temperatures for the Two Diag, Two Para and Tree Tria arrangements were staying in the medium level and showing very similar values throughout the test. 

#### 3.1.2. Effect of SMA Arrangement under RHE

The data for three different dependent variables (t_44_, t_56_ and T_fa_) are displayed in Table 2 to examine the effect of the SMA arrangement on TPP under the RHE condition. It was shown that all indices t_44_, t_56_ and T_fa_ were highly dependent on the SMA arrangement (*p* < 0.001). The fabric assembly with any SMA arrangement had a significantly higher t_56_ but lower T_fa_ compared with the CON (*p* < 0.05). The index t_56_ was only 36.4 s for the CON, while it increased by 23–81% due to the existences of the Two Diag, Two Para and Three Tria arrangements. The thermal protection time was much more improved when the One and the Three Diag were respectively incorporated with the fabric assembly, showing that temperatures at the internal surface of the thermal liner did not reach a criterion 56 °C after 70 s of RHE. In addition, the data from T_fa_ indicated that the One and the Three Diag were most effective in reducing the final temperature from 97.6 °C to a range of 42–44.9 °C. 

#### 3.1.3. Effect of Spring Size under RHE

**Figure 5:**
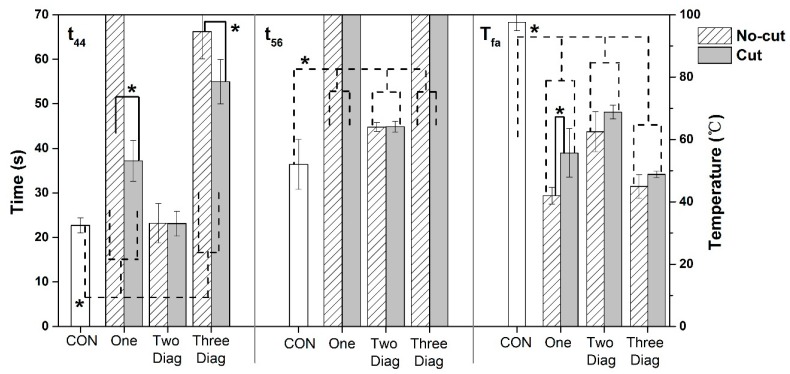
Comparisons of the thermal insulating property of the fabric assembly incorporated with two different sizes of SMA springs under the RHE condition (Note: * = significant difference was observed at *p* < 0.05. When t_44_ or t_56_ = 70 s, it means that temperatures did not reach 44 °C or 56 °C throughout the radiant heat exposure. CON = control group without any coil; One = one coil; Two Diag = two coils positioned diagonally; Three Diag = three coils positioned diagonally).

The results of t_44_, t_56_ and T_fa_ for different spring sizes under the RHE condition are displayed in Figure 5. Regardless of the SMA arrangement, t_56_ and T_fa_ were significantly changed as the spring size increased (*p* < 0.05). For example, t_56_ for the CON was 36.4 s, and it was substantially increased to approximately 45 s for the Two Diag with either a Cut or a No-cut size. In addition, t_56_ was further raised to over 70 s when both the Cut and No-cut sizes of the One and the Three Diag were incorporated within the fabric assembly. For the One arrangement, the significant effect of the spring size was observed from the indices t_44_ and T_fa_ (*p* < 0.05), showing that t_44_ was almost doubled and T_fa_ decreased by 13.7 °C when the spring size changed from the Cut to the No-cut. For the Three Diag arrangement, the significant effect of the spring size was only detected from t_44_ (*p* < 0.05), exhibiting a 20% increase as the spring size increased from the Cut to the No-cut. However, for the Two Diag arrangement, there were no significant differences in t_44_, t_56_ and T_fa_ between the two different sizes of SMA springs.

### 3.2. Performance under HSC

Similarly, to examine the performance of fabric assemblies with SMA springs incorporated within them under the HSC condition, three aspects including the temperature profiles, the effect of SMA arrangement, and the effect of SMA size are respectively shown in this section. 

#### 3.2.1. Temperature Profiles under HSC 

**Figure 6:**
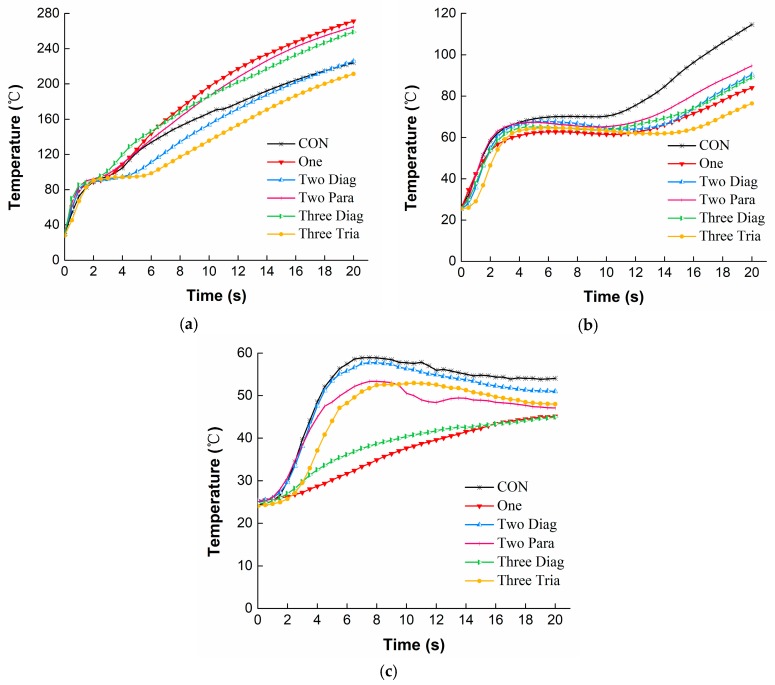
Temperatures for different arrangements of No-cut SMA spring under HSC: (**a**) The external surface of the moisture barrier; (**b**) The external surface of the thermal liner; (**c**) The internal surface of the thermal liner.

Figure 6 presents the temperature distribution through the fabric assemblies with different arrangements of No-cut SMA springs under the HSC condition. It can be seen from Figure 6a that all temperatures on the external surface of the moisture barrier increased immediately in the first 2 s when direct contacted with the hot surface. In addition, then the temperature was slightly changing before reaching a second quick rise in the following exposure. However, unlike the situation in Figure 4a showing that the CON under the RHE condition always had the lowest temperature on the external surface of the moisture barrier, under HSC this temperature ranked in the middle, showing higher values than the Two Diag and Three Tria but lower values than the One and Three Diag. In Figure 6b, all temperatures on the external surface of the thermal liner showed similar change trends. Temperature rose for the first 2 s of exposure, subsequently reached a relatively steady state during 2–12 s of exposure, and then increased sharply again until the end of exposure. As shown in Figure 6c, the temperature on the internal surface of the thermal liner was largely decreased compared to the temperatures on the external surfaces of the moisture barrier and the thermal liner. Temperatures observed for the One and the Three Diag increased more slowly throughout the exposure, showing an ending temperature approximated 45 °C. However, temperatures for other conditions rose initially to a range of 53–57 °C, and then decreased to 47–52 °C. 

#### 3.2.2. Effect of SMA Arrangement under HSC

As shown in Table 3, the indices t_44_ and T_fa_ were significantly affected by the SMA arrangement under the HSC condition (*p* < 0.001). The frequent appearances of NR in the t_56_ demonstrated that except for the CON and the Two Diag, fabric assemblies with other arrangements of SMA did not reach 56 °C after 20 s exposure to the HSC condition. The available data indicated that t_44_ and t_56_ under the HSC were much lower as compared to those under the RHE. For example, the maximum t_44_ listed in Table 3 was 17.7 s, which accounted for only 27% of that in Table 2. There was no significant difference existed in t_44_ among the CON, Two Diag, Two Para and Three Tria arrangements (*p* > 0.05). However, t_44_ increased nearly four times from 3.5 s to a range of 17.1–17.7 s when the One and the Three Diag were incorporated with the fabric assemblies. The index T_fa_ observed for the CON was only 54.1 °C, which was significantly reduced by the five different arrangements of SMA springs (*p* < 0.05). The difference in T_fa_ was particularly noticeable when the CON was compared to the One and the Three Diag. However, no significant difference was observed between the One and the Three Diag. 

#### 3.2.3. Effect of Spring Size under HSC

**Figure 7:**
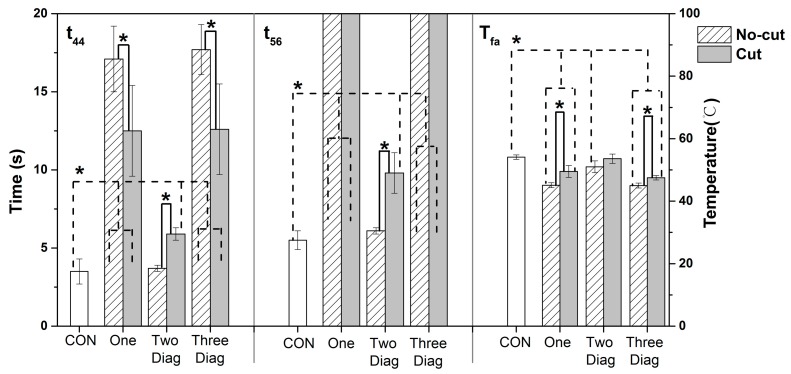
Comparisons of the thermal insulating property of the fabric assembly incorporated with two different sizes of SMA springs under the HSC condition (Note: * = significant difference was observed at *p* < 0.05. When t_56_ = 20 s, it means that temperatures did not reach 56 °C throughout the hot surface contact. CON = control group without any coil; One = one coil; Two Diag = two coils positioned diagonally; Three Diag = three coils positioned diagonally).

Figure 7 illustrates the TPP of the fabric assemblies with different sizes of SMA springs under the HSC condition. There were significant effects of SMA spring sizes on t_44_, t_56_ and T_fa_ (*p* < 0.05). For instance, the Cut size of the One and the Three Diag had a t_44_ of approximately 12.5 s, which was nearly three times higher as compared with the CON. In addition, their No-cut size had a much higher t_44_ reaching to 17.5 s. When the Cut size of the Three Diag was incorporated within the fabric, a significantly 12% decrease from 54 °C to 47.5 °C was found in T_fa_. In addition, this decrease was expanded to 17% as the No-cut size of the Three Diag was used. However, there was no significant difference in T_fa_ between the Cut and the No-cut size of the Two Diag (*p* > 0.05). Moreover, it was shown that t_44_ and t_56_ were respectively 5.9 s and 9.8 s for the Cut size of the Two Diag, which was significantly higher than those of the No-cut size (*p* < 0.05). 

## 4. Discussion

From temperature curves, it was found that under both RHE and HSC conditions the CON resulted in higher temperatures on the external and internal surfaces of the thermal liner in comparison to the fabric assemblies with any SMA arrangement incorporated within them. This was because the SMA springs, with an actuation temperature 45 °C, was expanded to form a steady air gap between the moisture barrier and the thermal liner when the fabric assembly was exposed to thermal hazards. The heat conductivity of steady air is 0.027 W/m/°C, which approximates one sixth of the fiber’s [28]. Heat radiation/conduction from thermal environments to the fabric layers was much impeded and heat loss by convection through the fabric layers increased due to the presence of the air gap, and thus the temperature of the thermal liner decreased. However, the results in Figure 4a showed that any arrangement of SMA springs under the RHE condition increased the external surface temperature of the moisture barrier. This could be explained by the fact that the activation of the SMA spring compressed the moisture barrier and decreased the distance between it and the radiant heating source, resulting in a higher temperature at the outside of this layer. Interestingly, in Figure 6a it was shown that the external surface temperature of the moisture barrier for the CON under the HSC condition was higher than that of the Two Diag and the Three Tria but lower than that of the One and the Three Diag. This observation on the external surface temperature of the moisture barrier under the HSC condition could be attributed to the arrangement of SMA spring and the location of the thermocouple for measuring the temperature. Both the One and the Three Diag had a SMA spring located in the center of the thermal liner, nicely forcing the temperature measuring point on the moisture barrier in approaching to the hot surface and thus increasing the external surface temperature of this fabric layer. However, arrangements of the Two Diag and the Three Tria did not have any spring located in the center of the fabric layer and the temperatures on their moisture barrier’s surfaces were not enhanced by the activation of SMA spring. 

Table 2 and Table 3 clearly showed that the SMA arrangement had significant influences on TPP of the fabric assemblies indicated by dependent variables of t_44_/t_56_ and T_fa_ under two different exposure hazards. The HSC condition resulted in a considerably lower t_44_ than the RHE condition, demonstrating that the direct physical contact between the fabric and a 400 °C hot surface caused greater heat transfer and less time to reach the criterion 44 °C as compared to the electromagnetic waves radiate from the quartz infrared lamps under the RHE condition. This quick heat transfer under the HSC condition caused the observation of three stages in the temperature profile in Figure 6c. An initially sharp increasing phase at the first 2 s of heat exposure was owing to the large temperature gradient between the fabric and the hot surface at the beginning of exposure. This large temperature gradient drove the heat transfer at a fast rate; consequently, the observed temperature increased rapidly. A second stable phase in about 12 s was due to the activation of the SMA spring. When the SMA spring was activated to open an air gap, heat convection through the air gap gradually counteracted the heat absorption of the fabric, and then an equilibration occurred in heat transfer. Upon further exposure, heat absorption of the fabric exceeded the heat convection, thereby showing a following increase in fabric temperature. The incorporation of any arrangement of SMA spring within the fabric assembly could improve the TPP to some extent, since the temperature at the end of exposure (T_fa_) was found to be notably reduced due to the use of SMA springs under both thermal exposures. The positive effect of the SMA spring under exposures to the RHE and HSC conditions consisted with those of Congalton’s study [18], in which the effectiveness of SMA springs was reported as the fabric was exposed to a radiant heat produced by a cone calorimeter. Under both RHE and HSC conditions, it was found that incorporations of the One and the Three Diag were the most effective way in reducing the internal temperature of the thermal liner (shown in Figure 4c and Figure 6c), increasing the thermal protection times of t_44_/t_56,_ and decreasing the final temperature T_fa_. The different contributions of the SMA arrangements to the TPP could be attributed to their open air gaps formed by the deformation of SMA springs.

To further examine the air gap effect, the details of the air gap distribution in different SMA arrangements are displayed in Table 4. The air gap between the moisture barrier and the thermal liner was uneven. The location where the spring was inserted had the biggest air gap, and the air gap was decreasing as the distance to the spring increased. Both the One and the Three Diag had one spring precisely located at the center of the fabric, creating an air gap there with a full size of 32 mm. Therefore, heat transfer through the central area was greatly impeded, and as a result temperatures there were largely decreased. However, for the Two Diag, Two Para and Three Tria arrangements the center of the fabric had no springs available, just showing smaller air gap sizes (27–31 mm) there. It was suggested that SMA springs should be inserted in a proper place of the protective clothing, especially where the thermal exposure attacks directly and usually. For example, the chest and forearm are suitable places because these parts are directly facing to a heat source when firefighters are holding a hose to extinguish a fire. Please note that the Three Diag had two more springs within the fabric layer as compared with the One, but it did not lead to a significantly higher thermal insulation when we examined the data of t_44_, t_56_ and T_fa_. It seemed to indicate that increasing the number of SMA springs did not show additional thermal insulation from the results of this study. This might be attributed to the limitations of the single-point temperature measurement method. As shown in Table 4, the Three Diag had a more uniform air gap than the One. This uniform air gap would improve better thermal insulation during a full-scale test in which an intact piece of protective clothing and a larger isolation area are measured, rather than one point in the middle of a small fabric sample tested in this study. This result should be confirmed by further studies. The use of multipoint temperature measurement or the infrared thermal camera technology that would provide a two-dimensional visible image of the temperature profile is suggested in future works to examine the potential of SMA springs as a suitable material for the development of intelligent garments. 

The results from Figure 5 and Figure 7 showed that TPP of fabrics was affected by the SMA spring sizes under both exposures to RHE and HSC. For the One and the Three Diag, there was a considerably increase (approximately 100–400%) observed in t_44_, even when a Cut size spring was incorporated within the fabric layers. This finding was consistent with those from previous researches, which have shown that the thermal protection against thermal exposures could be enhanced remarkably even though a small air gap size ranged 6–12 mm was added between the fabric layers [28,29]. The No-cut size of the One and the Three Diag resulted in a significantly higher t_44_ than the Cut size under both RHE and HSC conditions. This was because the No-cut springs were taller after they fully deformed in comparison to the Cut springs. This additional height increased the size of the air gap introduced between the moisture barrier and the thermal liner, thereby improving the thermal insulation of the fabric assembly. 

However, under the RHE condition no significant differences in t_44_, t_56_ and T_fa_ were detected between the Cut and No-cut size of the Two Diag. The likely reason that the significant effect of the SMA size was not observed for the Two Diag was the limitations of this spring arrangement. As can be seen from Table 4, the Two Diag had no springs located in the center of the fabric. The additional height of its No-cut size spring directly induced an air gap increase occurred where the two springs were inserted, but the air gap within the central area of the fabric could not increase as much as that resulted from the One and the Three Diag. This observation demonstrated that the impact of SMA spring size was affected by the arrangement mode, which could be related with the air gap shape caused by different SMA arrangements. Interestingly, under the HSC condition the No-cut size of the Two Diag had significantly lower t_44_ and t_56_ as compared to the Cut size. This result was opposed to those found under the RHE condition, i.e., the No-cut size of the spring increased the thermal protection time t_44_. A possible explanation for this might be the heat conduction caused by the SMA spring. In comparison to the RHE condition, physical contact under the HSC condition resulted in great heat transfer between the fabric and a hot surface. This great heat transfer increased the temperature of the spring and then increased the heat conduction from the copper-based spring to the fabric. Thus, the positive effect of the big spring size on reducing heat transfer was offset by the increasing heat conduction from the spring. The problem of heat conduction of the SMA spring was also motioned in a previous study, which have reported that the great conductive heat travelling through the spring is not benefit for the thermal protection [18]. It seemed to indicate that as compared to the RHE condition the HSC condition may add the risk of heat conduction from the metal-based SMA spring. An insulative disc with low heat conductivity might be used to attach the springs in the fabric, and thus decrease the heat conduction to skin [18]. In addition, this heat conduction should be paid more attention under the HSC condition because in real-life situations protective clothing with multilayers of fabric is easily compressed as firefighters are blundering into a structural heating wall, crawling on a hot ground, or holding a heated stuff. In these situations, the compressed protective clothing not only restricts the capacity of the air gap to open but also enhances heat conduction of the metal spring, all of which will limit the benefits of the SMA spring. Therefore, it is suggested that the SMA spring should not be placed in the elbow, knee, and back areas of the protective clothing, where the fabric layers are frequently squeezed if they are contacting with a hot surface. 

The above discussion demonstrated that the advantages of the SMA spring were achieved through providing additional air gaps between adjacent fabric layers as the SMA was activated by thermal exposures. In addition, the degree of this advantage depended on the size of the open air gap. Therefore, it was necessary to explore the specific corrections between the air gap size and the TPP of the fabric assembly. The regression analyses are suitable for estimating the relationship of TPP [23,27]. In this study, there were several NR shown in the columns of t_44_ and t_56_ in Table 2 and Table 3, meaning that these temperatures did not reach the criteria of 44 °C and 56 °C. The NR-labeled data were not suitable to use for the fitting analysis. Thus, the index T_fa_ that had every datum available was only chosen to conduct the fitting analysis. 

**Figure 8:**
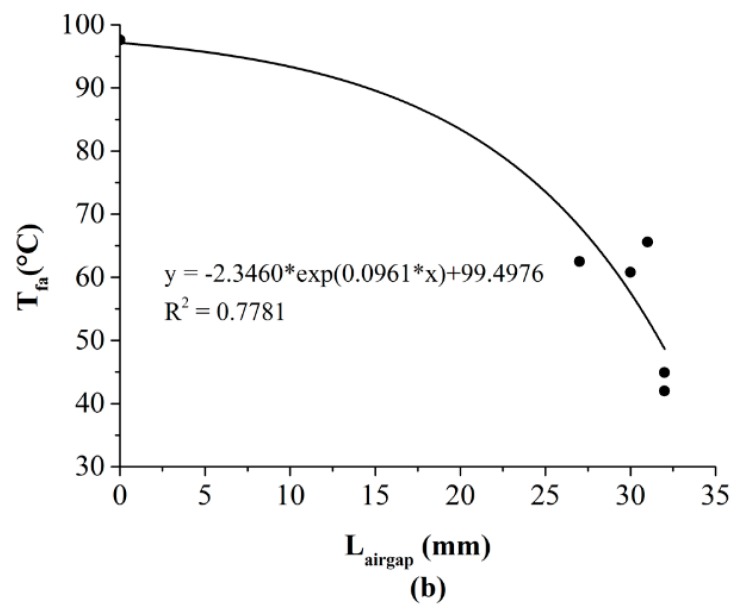
Relationship between the air gap size and the thermal protective performance under the RHE condition. (Note: RHE = radiant heat exposure; T_fa_ = final temperature; *L_airgap_* = air gap size).

**Figure 9:**
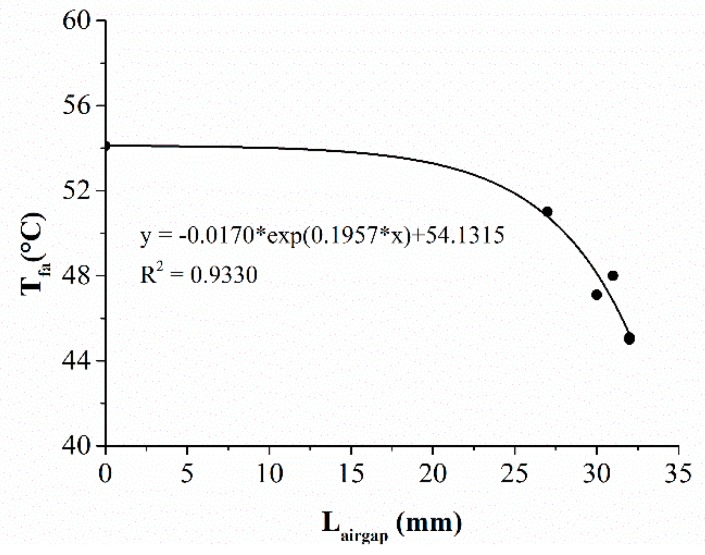
Relationship between the air gap size and the thermal protective performance under the HSC condition. (Note: HSC = hot surface contact; T_fa_ = final temperature; *L_airgap_* = air gap size).

The relationships between the air gap size and T_fa_ under the RHE and the HSC conditions are displayed in Figure 8 and Figure 9, respectively. Strongly negative exponential correlations with the air gap size were observed (RHE condition: R^2^ = 0.7781, HSC condition: R^2^ = 0.933). Equations (1) and (2) indicated that the final temperature of the fabric decreased with the increasing of the air gap size produced by the SMA spring. This meant that the increase of the open air gap size that was resulted from the different SMA arrangements and spring sizes had a positive effect on TPP of the fabric assembly. This was consistent with previous studies conducted on the conventional TPC that has no SMA springs incorporated within it [28,29]. If the conventional protective clothing has a bigger clothing size, heat transfer through the clothing is decreased due to the increasing air gap size between the clothing and the skin.

(1) $$T_{fa}=-2.3460\times \exp(0.0961\times L_{airgap})+99.4976$$

(2) $$T_{fa}=-0.0170\times \exp(0.1957\times L_{airgap})+54.1315$$

To further understand the effect of the air gap, heat transfer through the air gap was examined. When the air gap is formed due to the actuation of SMA springs, energy is transferred by both conduction/convection and radiation through the air gap. Assuming one-dimensional heat transfer of inclined channels through a plane fluid layer, the heat flux across the air gap (*q*) is given by [30]:

(3) $$q=q_{cond/conv,airgap}+q_{rad,airgap}$$

where *q_cond/conv,airgap_* refers to the heat flux by convection across the air gap, and *q_rad,airgap_* is the heat flux by radiation across the air gap. The heat flux by convection between the two adjacent fabric layers with an air gap is given as [30]:

(4) $$q_{cond/conv,airgap}=h_{c,airgap}(T_{a}-T_{b})$$

where *T_a_* and *T_b_* are the temperatures of the two adjacent clothing layers forming an air gap, *h_c,airgap_* is the convective heat transfer coefficient between the clothing layers, which is [30]:

(5) $$h_{c,airgap}=Nu\frac{k_{air}}{L_{airgap}}$$

where *Nu* is the Nusselt number, *k_air_* is the thermal conductivity of air, and *L_airgap_* is the size of air gap.

According to Torvi’s study [30], the radiation heat flux across the air gap is

(6) $$q_{rad,airgap}=\frac{\sigma (T_{a}{}^{4}-T_{b}{}^{4})}{\frac{1-\varepsilon_{a}}{\varepsilon_{a}}+\frac{1}{F}+\frac{1-\varepsilon_{b}}{\varepsilon_{b}}}$$

where *σ* is the Stefan-Boltzmann constant, *ε_a_* and *ε_b_* are the emissivity of the two clothing layers adjacent to the air gap, and *F* is the radiation view factor of two adjacent fabric layers, which is [31]:

(7) $$F=\frac{{\left(L_{airgap}^{2}+L_{fab}^{2}\right)}^{1/2}-L_{airgap}}{L_{fab}}$$

where *L_fab_* is the thickness of the fabric layer.

It can be seen from Equations (5) and (7) that the convective heat transfer coefficient *h_c,airgap_* and the radiation view factor *F* decrease with the increase of the air gap, and thereby reducing the convection and radiation across the air gap. It can be concluded that the air gap size that was influenced by the SMA spring arrangement and spring size has a positive effect on TPP, because the larger air gap size reduces the convective heat transfer coefficient and increases the view factor between the fabric layers.

## 5. Conclusions

In this study, SMA springs were incorporated into a three-layer firefighter turnout suit material in an endeavor to develop thermally responsive protective clothing that actuated to create extra air gap between the fabric layers under exposures to thermal hazards. The effects of the SMA arrangement mode and size on TPP of clothing were investigated under RHE and hot surface exposure. The incorporation of SMA springs within the fabric assembly could improve the TPP, but the extent of which the springs provided thermal protection was dependent on the arrangement and the size of the SMA springs. The effectiveness of reinforcing the firefighter protective clothing using the SMA springs depended on the capacity of the protective clothing to expand and form an air layer. For the effect of SMA arrangement, the One and Three Diag with No-cut springs were shown to be the most effective applications in enhancing thermal insulation, because they had one spring just located in the central of the fabric and created the biggest air gap there. It is suggested that SMA springs should be incorporated into the vulnerable parts of protective clothing where the thermal exposure directly and usually attacked (e.g., the chest, arms). 

For the effect of SMA spring size, there was a considerable increase of thermal insulation even if a Cut size of spring was incorporated within the fabric assembly, and the thermal insulation was further improved as the No-cut size of spring was used. One exception was that under the HSC condition the No-cut size of the Two Diag significantly decreased the thermal insulation as compared with its Cut size. This indicated that the HSC condition may add the risk of heat conduction travelling through the copper-based spring, while the benefit of the bigger spring size was pronounced under the RHE condition. In addition, relationships between air gap size and TPP under two different kinds of thermal exposures were successfully established using exponential equations. TPP increased with the increasing size of the open air gap. One way to improve the TPP of the intact protective clothing is to use a greater spring size to produce a bigger air gap size. In addition, a rational arrangement of SMA spring is also essential to insure the location and the shape of the air gap. The preferring locations of the produced air gap are the directly exposing areas of the protective clothing and increasing the number of SMA spring increases the uniformity of the air gap. The results of this study will guide the engineering of temperature-responsive protective clothing with SMA. 

## Figures and Tables

**Table 1:** Basic properties of the testing fabrics.

Layer	Component	Structural Features	Mass (g/m^2^)	Thickness (mm)
Outer shell	98% meta-aramid/2% para-aramid	Twill	193.7	0.49
Moisture barrier	100% meta-aramid/PTFE film	Water thorn felt with PTFE	108.3	0.85
Thermal liner	100% meta-aramid	Needle punched nonwoven with meta-aramid woven face cloth	200.0	0.72

Note: PTFE = polytetrafluoroethylene.

**Table 2:** Thermal insulating properties of the fabric assembly incorporated with No-cut SMA springs under the RHE condition.

Arrangement	Time to Reach 44 °C		Time to Reach 56 °C		Final Temperature	
	t_44_ [s] (SD)	The Ratio to CON	t_56_ [s] (SD)	The Ratio to CON	T_fa_ [°C] (SD)	The Ratio to CON
CON	22.7 (1.7) ^a^	1.00	36.4 (5.6)	1.00	97.6 (10.2)	1.00
One	NR	>3.08	NR	>1.92	42.0 (2.7) ^b^	0.43
Two Diag	23.2 (4.4) ^a^	1.02	44.8 (1.0)	1.23	62.5 (6.4)	0.64
Two Para	28.4 (4.1) ^a^	1.25	55.0 (5.9)	1.51	60.8 (4.7)	0.62
Three Diag	66.2 (6.1)	2.92	NR	>1.92	44.9 (3.8) ^b^	0.46
Three Tria	26.4 (3.5) ^a^	1.16	65.7 (6.4)	1.81	65.6 (3.8)	0.67
EA	**		***		***	

Note: SD = standard deviations; t_44_/t_56_ = the time to reach the temperature of 44 °C/56 °C; T_fa_ = the final temperature throughout the test; NR = the temperature does not reach the criterion of 44 °C or 56 °C; EA = the effect of the SMA arrangement; *** *p* < 0.001, ** *p* < 0.01. ^a^, ^b^—each testing sample with the same superscript letter do not differ significantly from each other (*p* > 0.05), otherwise significant differences determined between each sample using LSD post hoc tests (*p* < 0.05); CON = control group without any coil; One = one coil; Two Diag = two coils positioned diagonally; Two Para = two coils positioned in parallel; Three Diag = three coils positioned diagonally; Three Tria = three coils arranged at the vertices of an equilateral triangle.

**Table 3:** Thermal insulating properties of the fabric assembly incorporated with No-cut SMA springs under the HSC condition.

Arrangement	Time to Reach 44 °C		Time to Reach 56 °C		Final Temperature	
	t_44_ [s] (SD)	The Ratio to CON	t_56_ [s] (SD)	The Ratio to CON	T_fa_ [°C] (SD)	The Ratio to CON
CON	3.5 (0.8) ^a^	1.00	5.5 (0.6) ^a^	1.00	54.1 (0.7)	1.00
One	17.1 (2.1) ^b^	4.89	NR	>3.63	45.1 (0.8) ^a^	0.83
Two Diag	3.7 (0.2) ^a^	1.06	6.1 (0.2) ^a^	1.11	51.0 (1.9)	0.94
Two Para	3.9 (1.4) ^a^	1.11	NR	>3.63	47.1 (1.0) ^b^	0.87
Three Diag	17.7 (1.6) ^b^	5.06	NR	>3.63	45.0 (0.8) ^a^	0.83
Three Tria	5.0 (0.3) ^a^	1.43	NR	>3.63	48.0 (1.3) ^b^	0.89
EA	***		NS		***	

Note: SD = standard deviations; t_44_/t_56_ = the time to reach the temperature of 44 °C/56 °C; T_fa_ = the final temperature throughout the test; NR = the temperature does not reach the criterion of 44 °C or 56 °C; EA = the effect of SMA arrangements; *** *p* < 0.001; NS = no significant difference was observed at *p* > 0.05. ^a^, ^b^—each testing sample with the same superscript letter do not differ significantly from each other (*p* > 0.05), otherwise significant differences determined between each sample using LSD post hoc tests (*p* < 0.05). CON = control group without any coil; One = one coil; Two Diag = two coils positioned diagonally; Two Para = two coils positioned in parallel; Three Diag = three coils positioned diagonally; Three Tria = three coils arranged at the vertices of an equilateral triangle.

**Table 4:** Air gap distribution in different arrangements of No-cut SMA springs.

Arrangement	Diagrammatic Presentation	Air Gap Distribution Characters
One	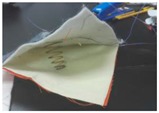	The center has the biggest air gap (32 mm), and the corner with no fixation has an air gap of approximately 9 mm.
Two Diag	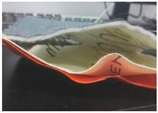	The location where the spring is inserted has the biggest air gap, but the center has a smaller air gap (27 mm). The corner with no fixation has an air gap of approximately 22 mm.
Two Para	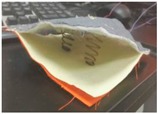	The location where the spring is inserted shows the biggest air gap, but the center has a smaller air gap (30 mm). The center of the edge that is in line with the two springs has a 26 mm air gap, while the center of the other edge has a 15 mm air gap.
Three Diag	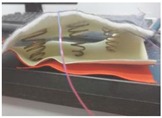	The air gap located in the line of the springs is becoming uniform. The spring at the center creates the biggest air gap of 32 mm, but the springs closer to the fixed corner create only 14 mm air gap. The corner with no fixation has an air gap of approximately 24 mm.
Three Tria	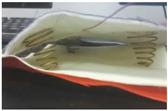	The air gap in an equilateral triangle area formed by the springs is becoming uniform. The air gap at the center is about 31 mm, and the air gap at the corner with no fixation is approximately 26 mm.

Note: CON = control group without any coil; One = one coil; Two Diag = two coils positioned diagonally; Two Para = two coils positioned parallelly; Three Diag = three coils positioned diagonally; Three Tria = three coils arranged at the vertices of an equilateral triangle.
